# Agency of schoolchildren: dataset collected using a new comprehensive multidomain tool

**DOI:** 10.3389/fpsyg.2026.1660530

**Published:** 2026-02-23

**Authors:** Pavel Sorokin, Mikhail Goshin, Dmitry Grigoryev, Tatiana Krasnova

**Affiliations:** 1 Laboratory for Human Capital and Education Research, Institute of Education, National Research University Higher School of Economics, Moscow, Russia; 2 Center for Socio-Cultural Research, National Research University Higher School of Economics, Moscow, Russia

**Keywords:** adolescent development, agency, agency assessment tool, child development, schoolchildren agency

## 1 Introduction

Rapid shifts in social reality, increasing instability in the labor market, culture and socio-political field, highlight the necessity for individuals to be proactive toward their environment (Cavazzoni et al., 2022). The concept of agency is central in this context, reflecting the capacity for independent decision making and related behavior aimed at transformation or active support of the surrounding social landscape (Sorokin and Froumin, 2022; Cavazzoni et al., 2022).

There are two key aspects of agency emphasized in the literature. The first pertains to an individual's ability to navigate social contexts and control their life while setting and achieving goals (Emirbayer and Mische, 1998; Cavazzoni et al., 2022). The second involves influencing one's social environment to transform and create new social structures (i.e. communities or forms of interaction Sorokin and Froumin, 2022). Agency entails moving beyond the current state of affairs (Hopwood and Sannino, 2023), which usually implies such stages as goal-setting, planning, action, and reflection (Emirbayer and Mische, 1998; Crockett, 2002; Archer, 2005).

In terms of goal-setting, two forms of agentic behavior may be identified, namely ego-oriented agency, focused exclusively on personal goals, and socially-oriented agency, aimed at increasing the wellbeing of others or bringing any kind of benefits to them (Chen et al., 2019). Under conditions of neo-structuration, when social structures are increasingly dependent upon individual agency, the latter may be seen as both an inherent human trait and a catalyst for social change, essential for addressing acute social problems (Sorokin and Mironenko, 2025). Recent studies on Russian schoolchildren have shown that agency related to personal and communal goals often coexists harmoniously (Goshin and Sorokin, 2024).

Although agency is widely discussed in literature, comprehensive measurement tools are still rare and highly diverse (Cavazzoni et al., 2022). Empirical research typically treats agency as an “umbrella term,” measuring related concepts such as autonomy and the ability to influence one's life, and the capacity to make choices (Zimmerman et al., 2019). Other proxies for agency include confidence (Veronese et al., 2019) along with planning and goal-setting (Bratman, 2022).

Currently, few comprehensive instruments measure agency of schoolchildren (Cavazzoni et al., 2022). Existing models focus on personal goals rather than contributions to social transformation or the proactive maintenance of social institutions (Donald et al., 2020). For instance, “agentic engagement” in education primarily addresses behavior within the formal school system (Reeve and Shin, 2020). This highlights the need for a broader tool to assess agency in various contexts where it manifests (Zimmerman et al., 2019).

Existing research conceptualizes agency across multiple domains such as family, school, and peer groups (Oswell, 2013; Gurdal and Sorbring, 2018).

However, the role of education in shaping agency is complex. On one hand, the rapid cognitive and emotional development during school years expands adolescents' “horizon of possibilities,” fostering greater autonomy and a clearer sense of personal goals, which correlates with increased agency manifestations (Nunes et al., 2025).

On the other hand, social contexts, including educational institutions, can significantly constrain opportunities for agentic expression (Abebe, 2019; Priestley et al., 2015). Research indicates that students often perceive teachers as figures of institutional authority that limit their agentic expression (Gurdal and Sorbring, 2018), and even structured activities like project-based learning can be dominated by teachers, curtailing genuine student initiative (Manyukhina, 2022). This highlights the difference between engagement (aimed at achieving system-defined goals) and agency (implying autonomy, choice, and freedom; Klemenčič, 2017).

Key areas of schoolchildren's agency include family (Oswell, 2013), educational settings (in-class and extracurricular; Burger and Walk, 2016), local social environments (peer relationships; Nunes et al., 2025), and increasingly, early labor involvement (Cavazzoni et al., 2022; Schoon and Heckhausen, 2019). Schoolchildren demonstrate their agency in different ways (Gurdal and Sorbring, 2018), such as proposing solutions to family issues, initiating educational changes, or suggesting activities with peers. Notably, contemporary youth's agency often involves financial activities such as earning money through digital tools (Staff et al., 2023). Agency manifestation generally increases with age (Nunes et al., 2025; Schoon and Cook, 2021); studies also show gender differences, with girls often displaying agency in informal friendships and boys in achievement and competition-related domains (Schoon and Cook, 2021; Korlat et al., 2022; Yount et al., 2020). Socioeconomic status (SES) also influences agency, as higher SES often provides more opportunities for development (Schoon and Cook, 2021; Schoon and Heckhausen, 2019; Kim and Lim, 2019).

The literature highlights various factors that influence or correlate with schoolchildren' agency, despite differing measurement tools. Strong academic performance, often associated with boosted confidence and competencies may correlate with enhanced agency, but an excessive focus on achievement may suppress it by promoting conformity (Gurdal and Sorbring, 2018). The ability to plan and set goals is also crucial (Emirbayer and Mische, 1998), as skilled planners are more likely to pursue goals actively. Finally, place of residence influences opportunities and constraints for expressing agency (Zhang et al., 2018).

Datasets for quantitative assessment of schoolchildren' agency are scarce and, as far as the authors know, are not publicly open. In order to fill this gap, this study presents the dataset and descriptive statistics on schoolchildren's agency across multiple domains: family, education, school life, peer interactions, and financial activities (earning money). The dataset is based on the Agency Multidomain Assessment Tool (AMAT) developed to measure the level of agency based on typical agentic behaviors of schoolchildren across outlined domains. Our instrument operationalizes these dimensions through domain-specific behavioral indicators, with goal-setting, planning, action, and reflection serving as proxy manifestations of the underlying agentic capacities. Accordingly, the primary aim of this study is to introduce the Agency Multidomain Assessment Tool (AMAT) dataset and present it as a psychometrically sound resource for measuring multidomain agency in schoolchildren.

## 2 Methods

The study uses the results of a survey of schoolchildren in grades 5–11 (*N* = 2015; *M*_age_ = 14.6, *SD* = 1.5; 62.4% female) conducted from May to December 2023 in 18 regions of Russia. Respondents ranged in age from 10 to 18. The sample of respondents is presented in Table 1.

**Table 1:** The structure of the sample of respondents.

**Characteristics**	***N***
**Type of settlement**	
A city with a population of more than 1 million people	54.1%, *N* = 933
A city with a population of 100,000 to 1 million people	10.6%, *N* = 183
A city with a population of less than 100,000 people, an urban-type settlement	27.4%, *N* = 474
Village	7.9%, *N* = 136
**Mother's education**	
General secondary education or lower	4.7%, *N* = 78
Elementary or secondary vocational education	31.3%, *N* = 522
Tertiary professional education	64.0%, *N* = 1,070
**Father's education**	
General secondary education or lower	5.1%, *N* = 80
Elementary or secondary vocational education	41.4%, *N* = 649
Tertiary professional education	53.5%, *N* = 840
**The grade in which the respondent studies**	
5th−6th	4.0%, *N* = 72
7th−8th	40.4%, *N* = 728
9th	24.7%, *N* = 446
10th−11th	30.9%, *N* = 557

The sample is representative of Russian schools. A random stratified selection of schools was made, taking into account their size as a characteristic of the socio-economic status of an educational organization.

Before conducting the study, the research group asked the parents to give consent for their children to participate in the study. Only those adolescents whose parents had completed the consent form were included in the study. Additionally, we obtained informed assent from the adolescents themselves. Only those adolescents who provided both parental consent and their own assent were included in the study. The survey took place during class hours in the computer classrooms. Participants were informed that their participation was voluntary and that they could withdraw at any time without any negative consequence. The questionnaires were completed online on the Alchemer platform (https://www.alchemer.com/). Each participant was assigned a unique identification number. The time required to complete the questionnaires did not exceed 40 min. A supervising teacher was present in the classroom throughout the survey.

## 3 Agency multidomain assessment tool

AMAT was developed to measure the level of agency based on typical agentic behaviors of schoolchildren across outlined domains. It is a novel instrument developed by the authors, informed by the existing theoretical literature (Emirbayer and Mische, 1998; Gurdal and Sorbring, 2018; Staff et al., 2023), qualitative data on Russian schoolchildren's proactive behavior (Goshin and Sorokin, 2024). To capture the nuances of each domain, a series of situations were described and the respondents were asked about the frequency of encountering such situations in their lives, their subjective stance and behavior in relation to these situations.

The formulations offered in a questionnaire were as follows:

1. “There have been situations in my life (if yes, how often) when I have tried to persuade adult members of my family to make certain decisions that were quite important for the whole family (Family).”
2. “There have been situations in my life (if yes, how often) when I have criticized or made suggestions to improve the learning process at school, e.g., I have suggested a topic of interest, or I have suggested diversifying the lessons with new forms of learning (for example, games, projects, etc.; Educational Process).”
3. “There have been situations in my life (if yes, how often) when I have criticized or made suggestions to staff about extracurricular aspects of school life, e.g., discussed with staff my ideas about changing school uniforms, school meals, daily routines, etc. (School Life Beyond Educational Issues).”
4. “There have been situations in my life (if yes, how often) when I have suggested to my peers to engage in a new type of interesting activity (for example, a new game, a new way of spending leisure time, a new project, etc.) and they have agreed (Peers).”
5. “There have been situations in my life (if yes, how often) when I have earned money on my own and by my own initiative (Money).”

The frequency of each situation was assessed using a Likert scale with the following options:

- 4—such situations occur very often (once per week or more often)
- 3—frequently (about 1–2 times a month)
- 2—occasionally (once every 2–3 months)
- 1—rarely (once every 6 months or less often)
- 0—There have never been such situations

Subsequently, an integral variable for overall agency, “AMAT Total Score,” was created. This variable was calculated as the sum of scores from the frequency assessment across all situations, ranging from 0 to 20 (*M* = 6, *SD* = 3.6).

In accordance with the literature (Emirbayer and Mische, 1998; Cavazzoni et al., 2022), goal-setting is identified as the first key aspect of agency. In terms of goal-setting, the central question is how much of an action is spontaneous, immediate or oriented on a strategic impact; it is also important, whether action is self or other-(community)-orientated. It is generally accepted in the literature on agency that the actor needs to plan agentic behavior in advance to achieve a goal (Emirbayer and Mische, 1998). Thus, the second key aspect of agency is the planning of corresponding operations and the construction of their sequence for goal attainment, including assessment of possible risks. Finally, based on the works of Emirbayer and Mische (1998) and Archer (2005), reflexivity is an important characteristic of agentic action (understood as comprehension of the obtained results and assessment of related efforts).

Regarding the situations in each of the five spheres of agency manifestation, the respondents were asked to rate to what extent did the related characteristics correspond to their actions.

Data analysis included performing a Confirmatory Factor Analysis (CFA) to evaluate the latent variable of agency (AMAT) and its indicators. Model fit was assessed using the following indices: relative chi-square (χ^2^), standardized root mean square residual (SRMR), root mean square error of approximation (RMSEA), and comparative fit index (CFI). Measurement invariance across gender and grade groups was examined using multigroup confirmatory factor analysis, focusing on configural, metric, and scalar invariance. Reliability was assessed using Cronbach's alpha (α) and McDonald's omega (ω).

## 4 Data description

### 4.1 Descriptive statistics

Our findings indicate that Russian schoolchildren most frequently demonstrate agency in interactions with peers and in the domain of earning money, while exhibiting it least often within the school context, concerning both academic processes and extracurricular activities.

The frequency of agency manifestation in various fields of life and the set of developed indicators for each of the five areas are presented in Table 2.

**Table 2:** Descriptive statistics.

**Field**	**There have never been such situations**	**Rarely (once every 6 month or less)**	**Occasionally (once every 2–3 month)**	**Frequently (about 1–2 times a month)**	**Very often (1 time per week or more often)**
**The frequency of agency manifestation in various fields, %**					
Family	16.9%	27.7%	29.2%	17.7%	8.6%
Educational Process	42.2%	23.8%	16.9%	12.0%	5.2%
School Life Beyond Educational Issues	55.5%	18.6%	11.1%	9.3%	5.5%
Peers	26.7%	19.0%	21.5%	20.4%	12.4%
Money	37.1%	21.6%	15.4%	13.7%	12.2%
**Descriptive statistics: frequency and percentage**					
**Question**	**Stage**	**Completely does not correspond**	**Rather does not correspond**	**Rather corresponds**	**Fully corresponds**
My goal was to influence a specific situation “here and now”	Goal setting (immediate impact orientation)	13.3%, *N* = 215	34.6%, *N* = 560	36.2%, *N* = 586	15.8%, *N* = 256
My goal was to change the behavior and views of someone around me	Goal setting (strategic impact orientation, collaborative)	9.0%, *N* = 145	25.0%, *N* = 404	48.2%, *N* = 780	17.8%, *N* = 288
When I took actions, I pursued my personal interests only	Goal setting (self-oriented)	11.3%, *N* = 183	33.1%, *N* = 536	39.5%, *N* = 639	16.0%, *N* = 259
When I took actions, I also pursued the interests of someone around me	Goal setting (other oriented)	10.2%, *N* = 165	28.6%, *N* = 463	47.7%, *N* = 771	13.5%, *N* = 218
I planned my actions in advance (yes/no)	Planning	6.0%, *N* = 97	20.7%, *N* = 334	44.3%, *N* = 716	29.1%, *N* = 470
I assessed the risks associated with the possible negative consequences of such behavior for me	Planning (risk assessment)	5.7%, *N* = 92	16.8%, *N* = 272	45.8%, *N* = 740	31.7%, *N* = 513
I managed to achieve my goals	Reflection (achieving the result)	4.0%, *N* = 64	13.3%, *N* = 215	55.8%, *N* = 902	27.0%, *N* = 436
Achieving the goal required additional efforts from me, which I did not initially expect	Reflection (achieving the result)	6.5%, *N* = 105	28.9%, *N* = 468	43.3%, *N* = 700	21.3%, *N* = 344

### 4.2 Measurement model

CFA was conducted to examine the relationships between the latent variable Agency and its indicators, which correspond to the five outlined above domains. The Diagonally Weighted Least Squares (DWLS) method was used for estimation, and optimization was performed using the Non-linear Minimization subject to Box Constraints (NLMINB) method. The model converged successfully after ten iterations, with a sample size of 1,945 and 25 degrees of freedom.

The evaluation of the single-factor model's quality showed excellent fit indices to the data: chi-square χ^2^_(5)_ = 10.5, *p* = 0.062; SRMR = 0.018; RMSEA = 0.024 (95% confidence interval: = 0.001, 0.044); and CFI = 0.999. All factor loadings were significant (*p* < 0.001) and ranging from 0.569 (educational process) to 0.826 (other aspects of school life), with an average factor loading of 0.652. This indicates a strong relationship between each component and the agency construct. Reliability analyses confirmed acceptable internal consistency for the Agency construct, with Cronbach's α = 0.74 and McDonald's ω = 0.75.

### 4.3 Measurement equivalence

A multigroup CFA was performed to test measurement equivalence across sex and class groups, assessing configural, metric, and scalar invariance. The analysis used the DWLS method. Changes in CFI (ΔCFI ≤ 0.01) served as the primary criterion for establishing equivalence.

For configural equivalence, both models demonstrated a good fit: for sex, CFI = 1.000; for class, CFI = 1.000. This indicates that configural equivalence is supported across all groups. When testing for metric equivalence, the CFI for sex was 0.996 (ΔCFI = 0.004), and for class, it was also 0.996 (ΔCFI = 0.004). Both ΔCFI values were ≤ 0.01, confirming metric equivalence. For scalar equivalence, the CFI for sex was 0.992 (ΔCFI = 0.004), and for class, it was also 0.992 (ΔCFI = 0.004), supporting scalar equivalence. In summary, configural, metric, and scalar equivalence were established across sex and class groups based on the ΔCFI ≤ 0.01 criterion.

Reliability analyses showed acceptable internal consistency for the Agency construct across groups. For males, Cronbach's α = 0.74 and McDonald's ω = 0.76. For females, Cronbach's α = 0.73 and McDonald's ω = 0.74.

Regarding the class groups, reliability indices were consistently good. For the 5th-6th grade group, Cronbach's α = 0.75 and McDonald's ω = 0.76. In the 7th-8th grade group, Cronbach's α = 0.72 and McDonald's ω = 0.74. For the 9th grade group, Cronbach's α = 0.75 and McDonald's ω = 0.76. In the 10th-11th grade group, Cronbach's α = 0.74 and McDonald's ω = 0.75.

Thus, the results of the multigroup CFA demonstrated that configural, metric, and scalar equivalence are maintained across all groups by sex and class of study, as confirmed by the ΔCFI ≤ 0.01 criterion. Reliability analysis revealed acceptable internal consistency of the agency construct across all studied groups, with Cronbach's α ranging from 0.72 to 0.75 and McDonald's ω from 0.74 to 0.76. These findings indicate the high reliability and invariance of the measurement tool used across different demographic groups, suggesting that the model can be confidently applied to other datasets.

### 4.4 Intercorrelations between agency domains

To explore the interrelations between the different domains of agency, we calculated Pearson correlation coefficients for all five AMAT domain scores. The resulting correlation matrix is presented in Table 3.

**Table 3:** Intercorrelations between the five domains of the agency multidomain assessment tool (AMAT).

**Domain**	**Family**	**Educational process**	**School life beyond educational issues**	**Peers**	**Money**
Family	1				
Educational process	0.306^**^	1			
School life beyond educational issues	0.385^**^	0.419^**^	1		
Peers	0.351^**^	0.277^**^	0.437^**^	1	
Money	0.349^**^	0.287^**^	0.433^**^	0.347^**^	1

*N* = 1,945. ^**^p < 0.001 (two-tailed).

All inter-domain correlations are positive and statistically significant (*p* < 0.001), providing further support for a common underlying construct of agency measured by the AMAT. However, the strength of these associations varies.

The strongest correlations were observed for the domain of School Life Beyond Educational Issues, particularly with the Educational Process (*r* = 0.419), Peers (*r* = 0.437), and Money (*r* = 0.433). This suggests that agency expressed in the extracurricular aspects of school life (e.g., influencing daily routines like school clothes, meals, etc.) is closely linked to agentic behavior in formal learning, peer interactions, and financial initiative. The domain of Educational Process appeared most loosely connected with other areas of agency manifestation; lowest correlation is found with the Peers domain (*r* = 0.277).

This pattern of differential correlations underscores that while the five domains converge to form a coherent general factor (as confirmed by the CFA), they also capture distinct, non-identical facets of schoolchildren's agency. The domains are not interchangeable; each contributes unique variance. For instance, a schoolchild might be highly agentic in influencing Family decisions (*r* with School Life = 0.385) but less so in the Educational Process. Consequently, while the “AMAT Total Score” is a reliable composite measure, *its usage should take into account possible meaningful individual or group differences in the particular subject areas of agency*. Researchers using this dataset are encouraged to consider both the total score and the pattern of domain-specific scores to gain a more nuanced and balanced understanding of agency manifestations.

## 5 Limitations

Several limitations of the study should be acknowledged. First, the reliance on self-reported data may introduce biases such as social desirability. Second, the cross-sectional design prevents causal inferences about the development of agency. Third, as the analysis of domain intercorrelations shows, the composite AMAT total score, while reliable, should be supplemented by considering possible differences in agency manifestation across different life contexts. Researchers should therefore consider domain-specific scores alongside the total. Finally, the findings are based on a sample from Russian schools, and their generalizability to other cultural contexts requires further validation.

## 6 Future research

Several avenues for future research on the presented database and beyond may be proposed. The dataset and the AMAT instrument provide a foundation for advancing the general methodological issues and the theoretical understanding of schoolchildren's agency.

First, future research using this dataset could employ Item Response Theory (IRT) analysis (Toland, 2014) to evaluate the differential weighting, difficulty, and discrimination parameters of each AMAT item. This would allow for the creation of a weighted aggregate score that more precisely reflects the construct of agency.

Second, latent profile analysis (LPA) could be applied to identify distinct subgroups of schoolchildren based on their patterns of agency across the five domains (e.g., “peer-focused,” “school-influencers,” “all-round agentic actors,” etc.). Interesting would be to reveal how such profiles correlate with external outcomes like academic achievement or wellbeing.

Third, Structural Equation Modeling (SEM; Ullman and Bentler, 2012) with domain scores as separate but correlated latent variables, rather than a single sum score, would enable testing complex hypotheses about how agency in one domain (e.g., family) predicts or mediates agency in another (e.g., school life).

Thus, the present dataset may be helpful in stimulating further research—both on the same dataset and on different samples but using the same measurement tool. Further research should continue to explore the diverse manifestations of agency across different contexts and populations, elucidating its impact on academic performance, social engagement, and overall wellbeing. Fostering agency in schoolchildren is not only vital for their personal development but also for cultivating a generation of empowered individuals capable of shaping a better future.

## Data Availability

The original contributions presented in the study are included in the article/supplementary material, further inquiries can be directed to the corresponding author.
